# Association between red blood cell distribution width and encephalitis based on the pediatric intensive care unit database: a cross-sectional study

**DOI:** 10.3389/fneur.2025.1562921

**Published:** 2025-09-19

**Authors:** Weichao He, Qilin Yang, Rui Jiang, Xinyu Yang, Xujie Zhang, Ruoyu Cao, Xiaojuan Liu, Shanshan Tong

**Affiliations:** ^1^Department of Pediatrics, Cangzhou Central Hospital, Cangzhou, Hebei Province, China; ^2^Department of Critical Care, The Second Affiliated Hospital of Guangzhou Medical University, Guangzhou, Guangdong Province, China; ^3^Department of Neonatology, Cangzhou Central Hospital, Cangzhou, Hebei Province, China; ^4^Blood Transfusion Research Laboratory, Cangzhou Central Blood Station, Cangzhou, Hebei Province, China; ^5^Chinese Medicine Hall, Cangzhou Central Hospital, Cangzhou, Hebei Province, China

**Keywords:** red blood cell distribution width (RDW), encephalitis, cross-sectional study, critically ill children, pediatric intensive care unit (PIC) database

## Abstract

**Background:**

Encephalitis is an inflammatory disease of the brain parenchyma that continues to affect populations worldwide, with high morbidity and risk of long-term sequelae. Early prediction of its occurrence is very important to improve the outcomes of the childhood encephalitis. However, the relationship between red blood cell distribution width (RDW) and encephalitis remains unclear. We aimed to explore the association between RDW and encephalitis using a Chinese Pediatric Intensive Care Unit (PICU) database.

**Methods:**

In a cross-sectional study, we analyzed a China-based PIC database spanning from 2010 to 2018. Children admitted to the PIC with encephalitis were included as participants to investigate the correlation between RDW and children encephalitis. Additionally, multifactorial logistic regression, restricted cubic spline analysis models and stratified analyses were utilized to evaluate this relationship.

**Results:**

A total of 10,185 participants were enrolled, among whom the encephalitis prevalence was 1.7% (173/10,185). Multivariate regression models revealed that encephalitis in Chinese children was significantly decreased by 18% with 1% increase in RDW after adjusting for all covariates (Adjusted OR = 0.82, 95% CI: 0.73–0.92). When the RDW was analyzed using quartiles. The adjusted OR (95% CI) of encephalitis for participants in the highest RDW Q4 (≥15.9%) were 0.44 (0.23–0.85), respectively. Compared with individuals with lower RDW Q1 ( ≤ 13%; *P* for trend = 0.011). The association between RDW and childhood encephalitis was stable in the different subgroups (*P* for interaction >0.05). Interaction analysis revealed no interactive role in the association between RDW and encephalitis of the childhood.

**Conclusions:**

Our study indicated that higher RDW independently associated with reduced encephalitis prevalence in critically ill Chinese children. Validation through multicenter prospective studies is warranted to establish RDW's clinical utility.

## Introduction

Neurological disorders are increasingly recognized as leading causes of mortality and disability worldwide (1). Encephalitis—a complex syndrome characterized by brain parenchymal inflammation causing neurological dysfunction—poses significant public health challenges through substantial morbidity, mortality, and long-term sequelae. Global incidence ranges from 3.5 to 7.4 per 100,000 individuals annually, rising to 16 per 100,000 in pediatric population (2, 3). Clinical manifestations include fever (within 72 h of presentation), generalized or partial seizures (excluding febrile seizures), and new-onset focal neurological deficits (4). Management of autoimmune encephalitis typically requires immunosuppressive therapies (corticosteroids, IVIg, and rituximab). Immune checkpoint inhibitor-associated encephalitis demonstrates potential for substantial neurological recovery with immunosuppression, though long-term sequelae may persist (5). Comprehensive care necessitates systematic longitudinal follow-up, particularly given the risk of endocrine dysfunction following pediatric CNS infections. Standardized multidisciplinary monitoring enables early detection of endocrine sequelae, optimizing long-term outcomes (6). Infectious and autoimmune encephalitis often manifest with neurological deficits or recurrent attacks, imposing significant socioeconomic burdens (7). However, despite routine investigations by many excellent clinicians, etiological diagnosis that could not be clarified is found in up to 60% of cases of the encephalitis (8, 9).

Red cell distribution width (RDW) quantifies erythrocyte volume heterogeneity, with elevated values (anisocytosis) indicating impaired erythropoiesis or underlying pathology (10). Beyond its traditional role in anemia classification, RDW independently predicts adverse outcomes across multiple disorders (11) and correlates with inflammatory, pro-apoptotic, and pro-fibrotic markers (12). Traditionally utilized in laboratory hematology for distinguishing between different types of anemias, RDW has accumulated increasing and compelling evidence associating anisocytosis with a variety of human disorders. Interestingly, RDW demonstrates strong associations with cardiovascular disease, thromboembolism, malignancy, diabetes, and organ failure—conditions significantly impacting population mortality (13–15). This evidence positions RDW as an inflammation-sensitive biomarker potentially predictive of neurological outcomes in pediatric populations.

However, the current evidence exploring the relationship between RDW and encephalitis remains limited. Therefore, this study endeavors to fill this knowledge gap by investigating the potential association between RDW and childhood encephalitis in critically ill children.

## Methods

### Data sources and study population

This cross-sectional study utilized the Pediatric Intensive Care (PIC) database—a pediatric-specific, deidentified repository containing comprehensive clinical records from the Children's Hospital, Zhejiang University School of Medicine. The PIC database encompasses 13,449 distinct hospital admissions involving 12,881 unique pediatric patients (aged 0–18 years) admitted to critical care units between 2010 and 2018 (16). Patients with multiple admissions, technical errors, or missing data were excluded.

PIC database is a public database. The present study was complied with the Declaration of Helsinki and was approved by the Institutional Review Board/Ethical Committee of the Children's Hospital, Zhejiang University School of Medicine (Hangzhou, China 2019_IRB_052) (16, 17). The institutional review board of CangZhou Central Hospital granted exemption due to use of deidentified public data, with waiver of informed consent. All protected health information was rigorously deidentified, ensuring confidentiality without impacting clinical care. Data access followed established protocols via the PIC website and PhysioNet, with a signed usage agreement. Throughout the entire research process, we maintained a sense of responsibility and adhered to the principles of cooperative research. Researcher Weichao He obtained access to the database and was involved in data extraction (Certification No. 41878202). More information regarding the data can be found on the PIC website (http://pic.nbscn.org/). Furthermore, our study findings were reported in accordance with the standards set by the Strengthening the Reporting of Observational Studies in Epidemiology (STROBE) guidelines (18).

### Study variables and outcome

#### Baseline red blood cell distribution width

RDW was calculated as the coefficient of variation (expressed as a percentage) using the formula: RDW = (Standard Deviation of Red Blood Cell Volume/Mean Corpuscular Volume) × 100 RDW = (Standard Deviation of Red Blood Cell Volume/Mean Corpuscular Volume) × 100, measured via automated hematology analyzers during routine complete blood count testing. Within the initial 24 h of admission, a standard blood test was employed to ascertain the RDW for each patient. This study focused on individuals with encephalitis who were admitted to Pediatric Intensive Care Unit (PICU).

#### Diagnosis of encephalitis

All participants in the study underwent lumbar puncture aspiration for confirmation of encephalitis diagnosis, as per established criteria (19), which includes assessment through multiple morphological and immunological analysis methods. These criteria encompassed the presence of encephalitis-related symptoms and signs, cerebrospinal fluid (CSF) analysis, electroencephalograph sensitivity and specificity, magnetic resonance imaging, as well as serological techniques and polymerase chain reaction. The study specifically focused on pediatric encephalitis patients admitted to intensive care units to explore the potential relationship between RDW and childhood encephalitis.

#### Covariates

This study included the following variables, selected based on existing literature and clinical expertise: sex (male or female), age (0–18 years old), race (Han ethnic, non-Han ethnic contained Yi ethnic, Tujia ethnic, Miao ethnic, Buyei ethnic ethic, Hui ethnic and others), ICU category, (CICU: cardiac intensive care unit; GICU: general intensive care unit; NICU: neonatal intensive care unit; PICU: pediatric intensive care unit; and SICU: surgical intensive care unit). Red blood cell distribution width (%), white blood cells (10^9^/L), red blood cells (10^9^/L), hemoglobin (g/L), platelet (10^9^/L), neutrophils (10^9^/L), lymphocyte count (10^9^/L), monocyte count (10^9^/L), alanine aminotransferase (ALT), aspartate aminotransferase (AST), albumin (g/L), bilirubin total (μmol/L), lactate dehydrogenase (LD; U/L), glucose (mmol/L), urea (mmol/L), creatinine (μmol/L), potassium (mmol/L), sodium (mmol/L), chloride (mmol/L), C-reactive protein (mg/dl), procalcitonin (ng/ml), fibrinogen (g/L), human IL-6 (pg/ml), ICU length of stay (LOS; This indicates is the length of stay for the patient for the given ICU stay), Hosptial LOS (his indicates is the length of stay for the patient for the given hospital stay), Hospital flag (This indicates whether the patient died within the given hospitalization. 1 indicates death in the hospital, and 0 indicates survival to hospital discharge). The 28-day mortality (This indicates whether the patient died within the 28 days. 1 indicates death in the hospital, and 0 indicates survival to hospital discharge). All laboratory data were obtained from measurements taken within the first 24 h of admission.

### Statistical analysis

Descriptive analysis was performed for all participants. Data are presented as mean ± standard deviation (SD) or median (interquartile range, IQR) for continuous variables and as frequency or percentage (*n*, %) for categorical variables. The differences in continuous and categorical variables were investigated using the independent and chi-squared tests, respectively. The odds ratios (ORs) and 95% confidence intervals (CIs) for encephalitis with RDW were determined using multivariate logistic regression models.

Multivariate Cox regression analysis was conducted by incorporating variables with a significance level of *P* < 0.05 in the univariate model and covariate screening. Covariate adjustments were carried out using the following models: Model I was adjusted for age and sex; Model II was adjusted for age, sex, ethnicity/race, ICU category, and hospital length of stay (LOS); Model III was adjusted for all factors with *P* values less than 0.05 in the univariate analysis and covariate screening (Supplementary Table S3), including age, sex, ethnicity/race, ICU category, hospital LOS, white blood cell count, red blood cell count, hemoglobin concentration, platelet count, albumin concentration, and total bilirubin concentration.

In addition, restricted cubic spline (RCS) regression was constructed with four knots positioned at the 5th, 35th, 65th, and 95th percentiles of RDW to assess the potential linear relationship between RDW and childhood encephalitis after adjusting for the variables in Model III.

Furthermore, we conducted subgroup heterogeneity analysis using multivariate logistic regression and assessed interactions between subgroups and RDW through likelihood ratio testing. To ensure the robustness of our findings, we also performed sensitivity analysis. RDW was treated as a categorical variable in the logistic regression models, with a trend test conducted and the first quartile (Q1 ≤ 13%) established as the reference. Acknowledging the potential impact of hemoglobin levels on RDW, subgroup analysis was conducted using stratified logistic regression models to explore the association between RDW and encephalitis in Chinese children, considering factors such as age (< 1 year, ≥1 year), sex (female, male), ethnicity (Han, others), white blood cell count (< 10^9^/L, ≥10^9^/L), hemoglobin level (< 90 g/L, ≥90 g/L), and albumin level (< 36 g/L, ≥36 g/L). The test for interaction in the logistic regression model was utilized to compare odds ratios (ORs) between the analyzed subgroups. In instances where the missing data variable exceeded 10%, multiple imputations were employed to address missing data for the covariates.

Given that the determination of the sample size was exclusively reliant on the provided data, no *a priori* statistical power estimates were conducted. All the analyses were performed with the statistical software packages R (http://www.R-project.org, The R Foundation) and Free Statistics software versions 1.8. A two-tailed test was performed, and *P* < 0.05 was considered statistically significant.

## Results

### Study population

**Figure 1:**
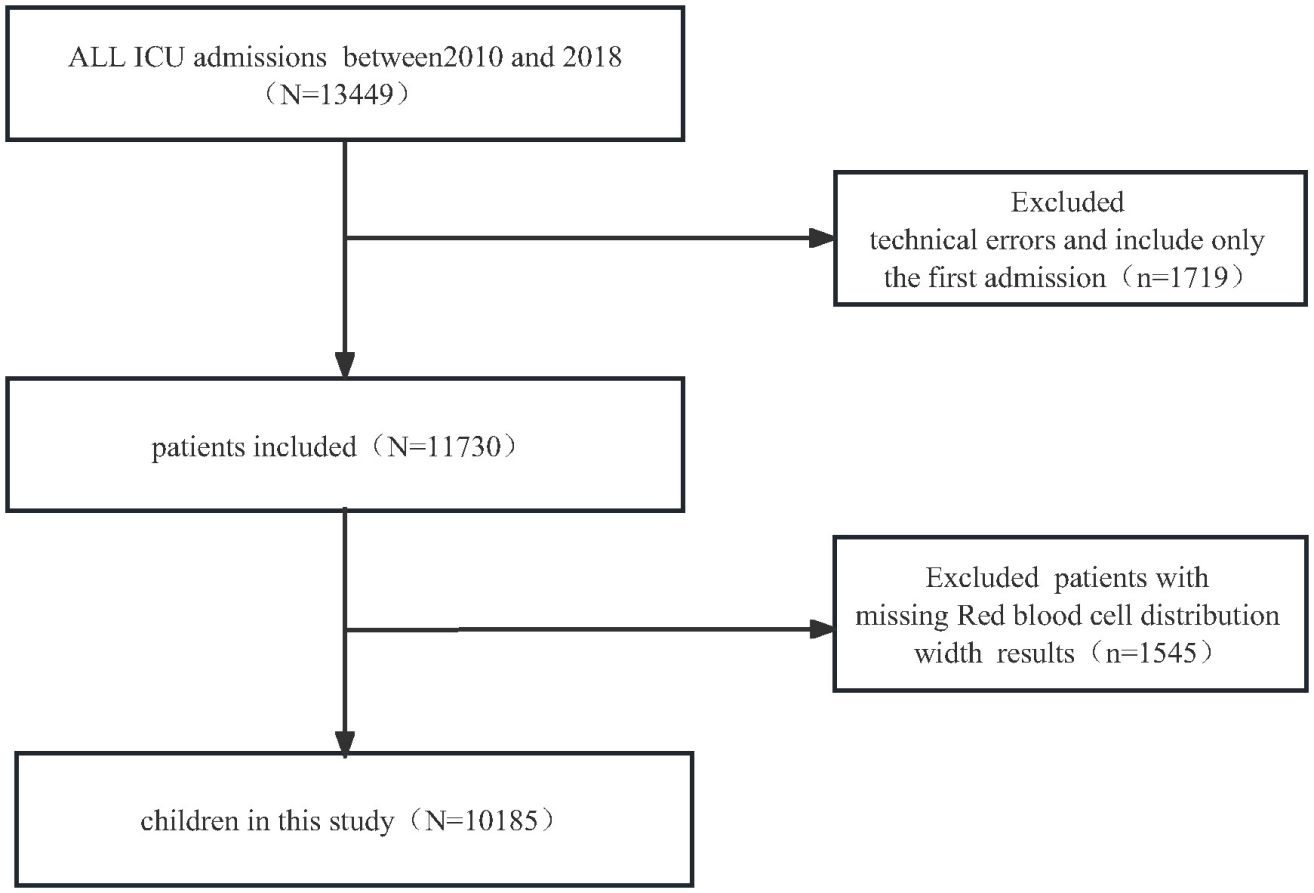
Flow chart of PICU admissions for this observational study.

For the retrospective cross-sectional study designed to examine encephalitis among patients with RDW, the PIC database included 13,449 distinct hospital admissions (aged 0–18 years) admitted to the PICU between 2010 and 2018. We first excluded 1,719 children with technical errors and patients with multiple admission, we further excluded 1,529 patients missing RDW results. Ultimately, a total of 10,185 pediatric patients were enrolled in the present study. The detailed inclusion and exclusion processes are illustrated in Figure 1.

### Baseline characteristics

Baseline characteristics of the 10,185 enrolled participants (5,803 boys and 4,382 girls) stratified by RDW quartiles are shown in Table 1. Median age at ICU admission was 0.7 years (0.2–3.2), median length of stay in ICU was 3.3 (1.0–11.1) days, median length of stay in hospital was 13.8 (7.9–23.5) days. The median RDW is 14.8% ± 2.5. Generally, the encephalitis prevalence in the present study was 1.7% (173 patients), which was, respectively 3.5% (85 patients), 2% (49 patients), 1% (25 patients), and 0.5% (14 patients) in RDW quartile 1–4 (*P* < 0.05). Participants in the highest quartile of the RDW (Q4 ≥ 15.9) were primarily female and younger individuals of Han ethnicity/race. Notably, they exhibited significantly lower levels of platelet count, albumin, alanine aminotransferase, glucose, C-reactive protein, fibrinogen, the presence of encephalitis and higher levels of white blood cells, red blood cells, hemoglobin, monocyte, lymphocyte, aspartate aminotransferase, bilirubin total, creatinine, urea, potassium, chloride, procalcitonin, interleukin-6, and hospital length of stay compared to individuals in the lowest RDW quartile (all *P* < 0.05).

**Table 1:** Baseline characteristics of patients from pediatric intensive care unit database 2010–2018 by categories of RDW levels.

**Variables**	**Total**	**Red blood cell distribution width quartiles (%)**				***P-*value**
		**Q1** ≤ **13**	**Q2 (13.1–14.2)**	**Q3 (14.3–15.8)**	**Q4** ≥**15.9**	
Participants	10,185	2,443	2,481	2,590	2,671	
Age, (year) Median (IQR)	0.7 (0.2, 3.2)	3.2 (1.1, 7.2)	1.4 (0.4, 3.9)	0.2 (0.1, 1.0)	0.2 (0.1, 0.8)	< 0.001
Sex, *n* (%)						< 0.001
Female	4,382 (43.0)	1,171 (47.9)	1,104 (44.5)	1,017 (39.3)	1,090 (40.8)	
Man	5,803 (57.0)	1,272 (52.1)	1,377 (55.5)	1,573 (60.7)	1,581 (59.2)	
Ethnicity, *n* (%)						< 0.001
Han	10,083 (99.0)	2,430 (99.5)	2,467 (99.4)	2,567 (99.1)	2,619 (98.1)	
Others^*^	102 (1.0)	13 (0.5)	14 (0.6)	23 (0.9)	52 (1.9)	
ICU category, *n* (%)						< 0.001
NICU	2,710 (26.6)	8 (0.3)	165 (6.7)	1,081 (41.7)	1,456 (54.5)	
GICU	1,360 (13.4)	624 (25.5)	297 (12)	213 (8.2)	226 (8.5)	
PICU	1,585 (15.6)	361 (14.8)	512 (20.6)	381 (14.7)	331 (12.4)	
SICU	2,316 (22.7)	701 (28.7)	787 (31.7)	484 (18.7)	344 (12.9)	
CICU	2,214 (21.7)	749 (30.7)	720 (29)	431 (16.6)	314 (11.8)	
WBC (10^9^/L), Median (IQR)	9.9 (6.8, 14.4)	9.6 (6.8, 13.4)	9.3 (6.3, 13.6)	9.8 (6.7, 14.3)	11.1 (7.3, 16.6)	< 0.001
RBC (10^12^/L), Mean ± SD	3.9 ± 0.8	4.0 ± 0.7	3.8 ± 0.7	3.9 ± 0.8	4.1 ± 1.1	< 0.001
HGB (g/L), Mean ± SD	116.9 ± 31.4	111.6 ± 18.7	107.8 ± 20.1	119.8 ± 31.8	127.4 ± 43.2	< 0.001
Monocyte (10^9^/L), Median (IQR)	0.4 (0.2, 0.8)	0.3 (0.2, 0.5)	0.3 (0.2, 0.6)	0.5 (0.3, 1.0)	0.7 (0.3, 1.2)	< 0.001
Lymphocyte (10^9^/L), Median (IQR)	2.4 (1.5, 3.5)	2.0 (1.3, 2.9)	2.2 (1.5, 3.2)	2.5 (1.6, 3.7)	2.8 (1.8, 4.2)	< 0.001
Neutrophils (10^9^/L), Median (IQR)	6.2 (3.6, 10.0)	6.8 (4.1, 10.4)	6.0 (3.5, 9.6)	5.7 (3.5, 9.6)	6.2 (3.4, 10.3)	< 0.001
PLT (10^9^/L), Mean ± SD	268.6 ± 143.5	287.9 ± 128.3	272.9 ± 139.7	266.4 ± 148.6	249.1 ± 152.2	< 0.001
Albumin (g/L), Mean ± SD	35.8 ± 6.2	38.5 ± 5.5	37.4 ± 5.8	34.6 ± 6.0	33.1 ± 6.2	< 0.001
ALT (U/L), Median (IQR)	17.0 (11.0, 31.0)	18.0 (12.0, 29.0)	20.0 (13.0, 33.0)	16.0 (10.0, 30.0)	15.0 (8.0, 31.0)	< 0.001
AST (U/L), Median (IQR)	45.0 (29.0, 82.0)	37.0 (26.0, 60.0)	46.0 (29.0, 84.0)	47.5 (30.0, 85.0)	53.0 (31.0, 99.0)	< 0.001
Bilirubin total (μmol/L), Median (IQR)	13.1 (7.2, 44.6)	8.7 (5.8, 12.5)	10.3 (6.3, 17.7)	24.4 (9.2, 86.2)	41.6 (12.4, 100.0)	< 0.001
Glucose (mmol/L), Median (IQR)	3.8 (2.9, 4.9)	4.1 (3.5, 5.0)	4.0 (3.2, 5.1)	3.7 (2.8, 5.0)	3.3 (2.4, 4.6)	< 0.001
LDH (U/L), Median (IQR)	400.0 (287.0, 599.0)	312.0 (241.0, 425.0)	372.0 (273.0, 529.0)	447.0 (322.0, 649.8)	514.0 (348.0, 776.0)	< 0.001
Creatinine (μmol/L), Median (IQR)	43.0 (35.0, 61.0)	41.0 (34.0, 50.0)	39.5 (33.0, 48.2)	45.0 (36.0, 68.0)	55.0 (38.0, 83.0)	< 0.001
Urea (mmol/L), Median (IQR)	3.5 (2.4, 4.8)	3.3 (2.4, 4.3)	3.2 (2.3, 4.4)	3.5 (2.4, 5.1)	3.8 (2.7, 5.6)	< 0.001
Potassium (mmol/L), Mean ± SD	3.8 ± 0.8	3.6 ± 0.7	3.7 ± 0.7	3.9 ± 0.8	4.0 ± 0.9	< 0.001
Sodium (mmol/L), Mean ± SD	137.2 ± 5.2	137.9 ± 4.8	137.8 ± 5.2	136.7 ± 5.2	136.5 ± 5.4	< 0.001
Chloride (mmol/L), Mean ± SD	108.9 ± 6.9	108.7 ± 5.8	108.6 ± 5.8	108.9 ± 6.1	109.5 ± 9.0	< 0.001
CRP (mg/dl), Median (IQR)	9.0 (4.0, 39.9)	11.0 (4.0, 45.9)	13.4 (4.0, 44.0)	8.0 (4.0, 36.1)	8.0 (4.0, 30.0)	< 0.001
PCT (ng/ml), Median (IQR)	0.4 (0.1, 1.6)	0.2 (0.1, 1.0)	0.4 (0.1, 1.5)	0.5 (0.1, 2.2)	0.5 (0.2, 2.2)	< 0.001
IL6, Median (IQR)	27.4 (8.6, 95.5)	22.8 (6.7, 74.5)	28.2 (8.2, 99.3)	28.6 (9.1, 108.9)	37.1 (11.8, 135.2)	< 0.001
Fibrinogen (g/L), Mean ± SD	2.3 ± 1.1	2.5 ± 1.0	2.3 ± 1.0	2.2 ± 1.1	2.0 ± 1.1	< 0.001
RDW (%), Mean ± SD	14.8 ± 2.5	12.4 ± 0.5	13.6 ± 0.3	15.0 ± 0.5	18.0 ± 2.5	< 0.001
Encephalitis, *n* (%)	173 (1.7)	85 (3.5)	49 (2)	25 (1)	14 (0.5)	< 0.001
LOS (day), Median (IQR)	3.3 (1.0, 11.1)	1.3 (0.9, 4.8)	2.0 (0.9, 6.0)	4.9 (1.6, 15.1)	8.3 (2.0, 26.8)	< 0.001
Hospital LOS (day), Median (IQR)	13.8 (7.9, 23.5)	10.1 (6.6, 16.0)	12.1 (7.7, 18.4)	15.9 (9.2, 27.6)	18.6 (9.5, 35.4)	< 0.001
In-hospital mortality, *n* (%)	590 (5.8)	90 (3.7)	105 (4.2)	155 (6)	240 (9)	< 0.001

Data are shown as the mean ± SD or median (IQR) for skewed variables or numbers (proportions) for categorical variables.

^*^Others ethnic groups include the Hui ethnic, baiyue ethnic, miao ethnic, tujia ethnic, yi ethnic, other ethnic.

Q, quartiles; RDW, Red blood cell distribution width; CICU, cardiac intensive care unit; GICU, general intensive care unit; NICU, neonatal intensive care unit; PICU, pediatric intensive care unit; SICU, surgical intensive care unit; WBC, White blood cells; RBC, Red blood cells; HGB, Hemoglobin; PLT, Platelet; ALT, Alanine aminotransferase; AST, Aspartate aminotransferase; LDH, Lactate dehydrogenase; CRP, C-reactive protein; PCT, procalcitonin; IL6, interleukin-6; Los, The length of stay for the patient for the given ICU stay which may include one or more ICU units. Hospital LOS, The length of stay for the patient for the given hospital stay.

### Association between RDW and the encephalitis

Regression analysis was performed to identify factors in this population that were associated with encephalitis. The results of univariate regression analysis indicated that age, ethnicity, RBC, lymphocyte, ALB ALT, AST, bilirubin total, chloride, fibrinogen, and hospital LOS, were positively associated with encephalitis (all *P* < 0.05, Supplementary Table S1).

Table 2 presents the association between RDW and encephalitis in multiple regression model. The prevalence of encephalitis was 1.7% (173/10,185). Overall, in the crude model and all adjusted models (Model I–III), the risk of encephalitis reduced as the quartile of RDW increased. Multivariate regression models revealed that encephalitis in Chinese children was significantly decreased by 18% with 1% increase in RDW after adjusting for all covariates (Model III, adjustment for age, gender, race, ICU category, white blood cell count, red blood cell count, hemoglobin level, platelet count, albumin level, and total bilirubin; Adjusted OR =0.82, 95% CI: 0.73–0.92). When the RDW level was analyzed using quartiles into four equal groups, there was also a significant inverse association between RDW and childhood encephalitis after adjusting for potential confounders. In the fully adjusted Model III, compared with lower RDW (Q1 ≤ 13%), the adjusted odds ratios (OR; 95% CI) for RDW and encephalitis participants in Q2 (13.1–14.2), Q3 (14.3–15.8), and Q4 (≥15.9) were 0.79 (0.54–1.17), 0.69 (0.42–1.14), and 0.44 (0.23–0.85; *P* for trend = 0.011), respectively. The association between RDW and childhood encephalitis was stable in the different subgroups (*P* for interaction >0.05).

**Table 2:** Multivariate regression analysis of association between RDW and the presence of encephalitis.

**Variable**	**n. encephalitis %**	**Non-adjusted**		**Model I**		**Model II**		**Model III**	
		**OR (95%CI)**	***P*** **value**	**OR (95%CI)**	***P*** **value**	**OR (95%CI)**	***P*** **value**	**OR (95%CI)**	***P*** **value**
RDW (%)	173 (1.7)	0.64 (0.57–0.71)	< 0.001	0.75 (0.67–0.83)	< 0.001	0.77 (0.7–0.85)	< 0.001	0.82 (0.73–0.92)	< 0.001
**RDW quartile**									
Q1 ≤ 13	85 (3.5)	1 (Ref)		1 (Ref)		1 (Ref)		1 (Ref)	
13.1 < Q2 ≤ 14.2	49 (2)	0.56 (0.39–0.8)	0.001	0.77 (0.53–1.11)	0.158	0.69 (0.47–1)	0.053	0.79 (0.54–1.17)	0.244
14.3 < Q3 ≤ 15.8	25 (1)	0.27 (0.17–0.42)	< 0.001	0.5 (0.31–0.81)	0.004	0.52 (0.32–0.84)	0.007	0.69 (0.42–1.14)	0.148
Q4 ≥ 15.9	14 (0.5)	0.15 (0.08–0.26)	< 0.001	0.27 (0.15–0.49)	< 0.001	0.28 (0.15–0.5)	< 0.001	0.44 (0.23–0.85)	0.015
*P* for trend	173 (1.7)		< 0.001		< 0.001		< 0.001		0.011

Q, quartiles; OR, odds ratio; CI, confidence interval; Ref, reference; RDW, red blood cell distribution width; WBC, white blood cells; RBC, red blood cells; HGB, hemoglobin; PLT, platelet.

Model I: adjusted by age+ gender.

Model II: adjusted by Model I+ ethnicity+ ICU category + hospital LOS.

Model III: adjusted by Model II + WBC+RBC+ HGB+PLT +Albumin +bilirubin total.

**Figure 2:**
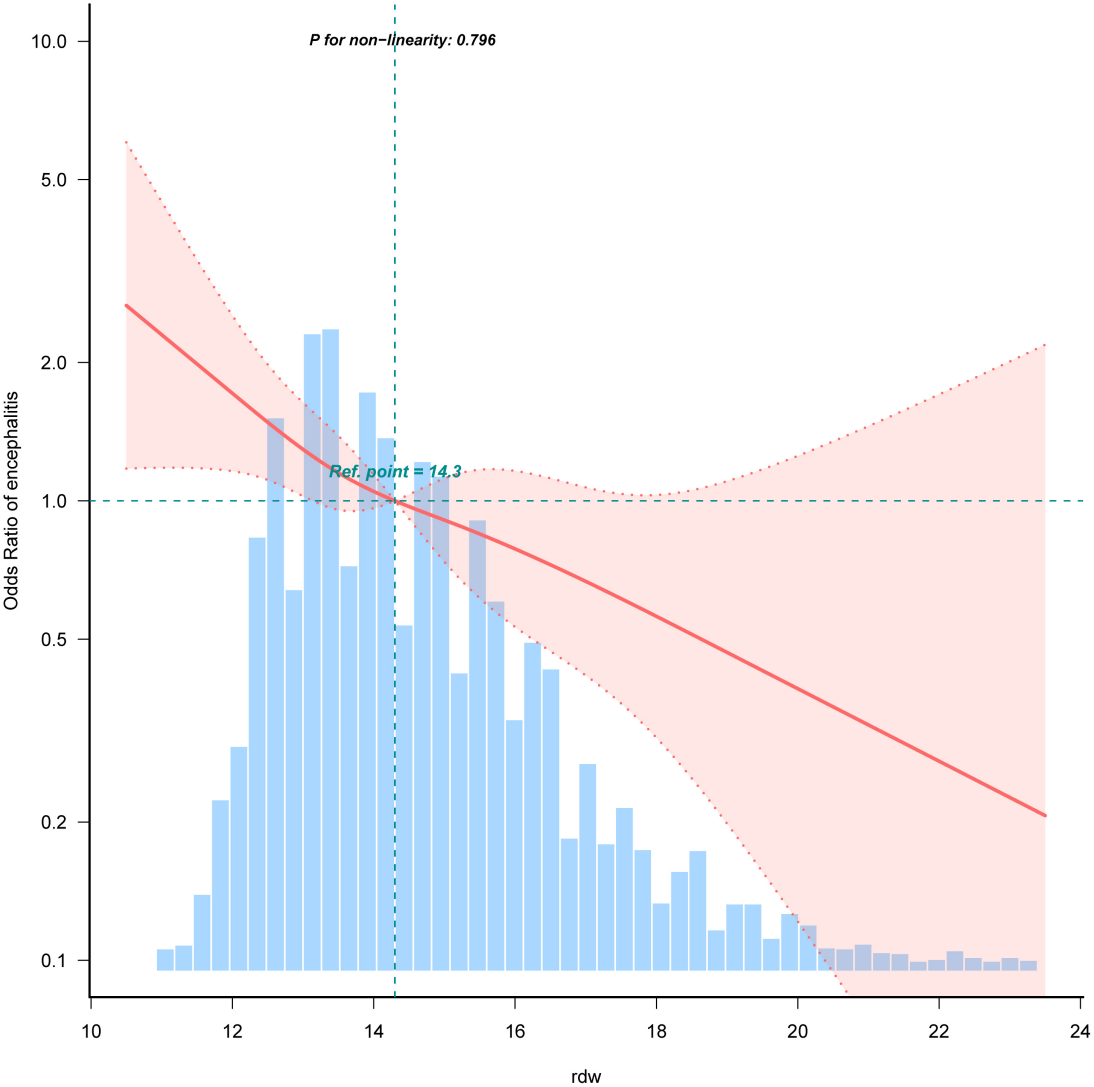
Restricted cubic spline analysis for RDW and childhood encephalitis. Association between RDW and odds ratio of the childhood encephalitis. Solid and dashed lines represent the predicted value and 95% confidence intervals. They were adjusted for age, sex, ethnicity, ICU category, hospital LOS, white blood cells, red blood cells, hemoglobin, platelet, albumin, and bilirubin total. Only 99% of the data is shown.

Smooth curve fitting (restrictive cubic spline) analysis accurately described a linear correlation between RDW and childhood encephalitis on the Figure 2. Our study findings revealed a significant negative association between RDW and the occurrence of encephalitis among Chinese children. They were adjusted for age, sex, ethnicity, ICU category, hospital LOS, white blood cells, red blood cells, hemoglobin, platelet, albumin, and bilirubin total. Only 99% of the data is shown.

### Subgroup analyses

**Figure 3:**
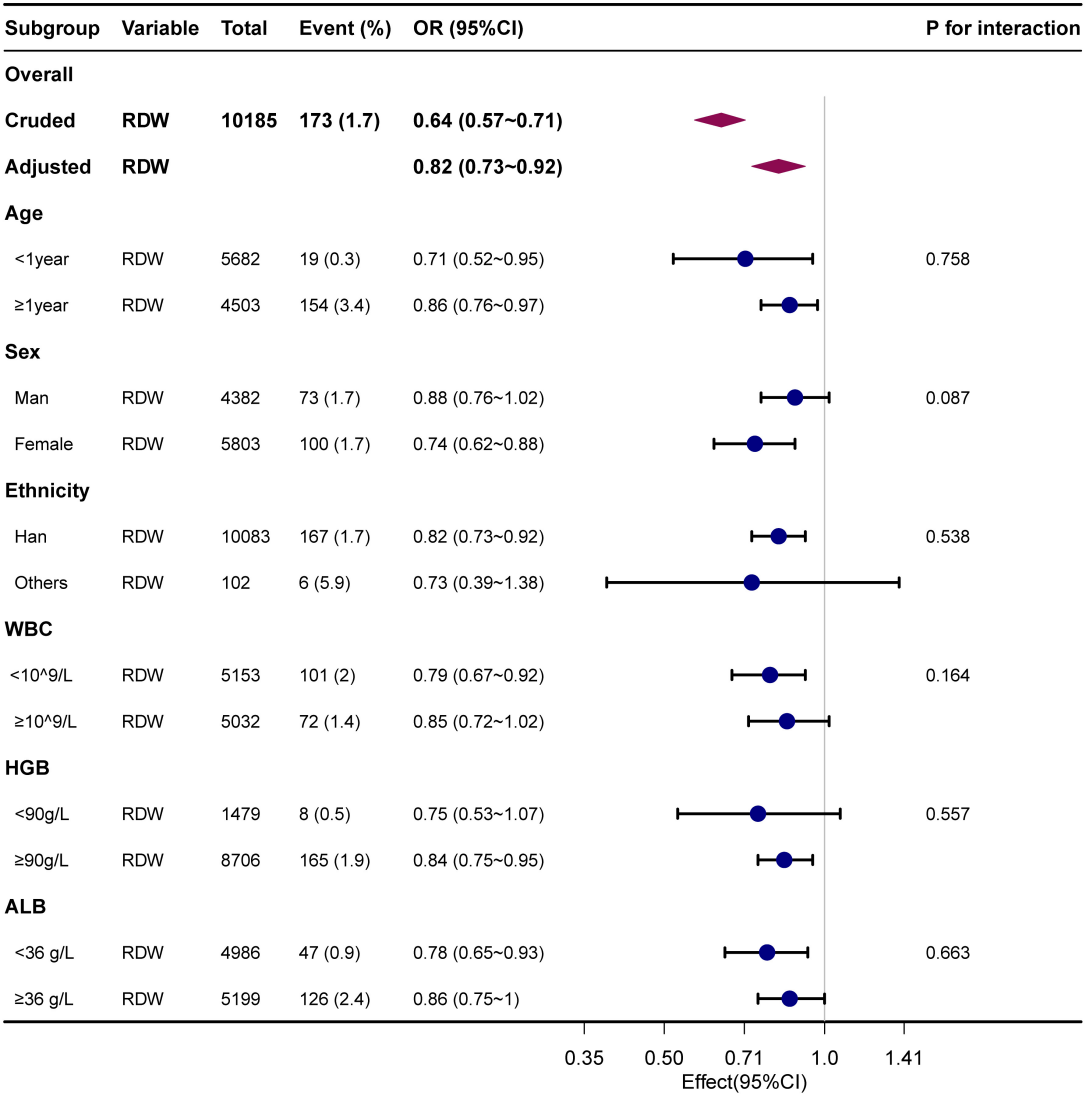
Forest plot for relationship between RDW and childhood encephalitis. Dots indicate odds ratios (ORs), with horizontal lines indicating 95% CIs. Diamonds indicate overall ORs, with outer points of the diamonds indicating 95% CIs. They were adjusted for age, sex, ethnicity, ICU category, hospital LOS, white blood cells, red blood cells, hemoglobin, platelet, albumin, and bilirubin total. Only 99% of the data is shown.

To explore the potential effect modification of various factors on the association between serum red cell distribution width (RDW) and encephalitis, we conducted subgroup and interaction analyses while adjusting for confounders including age, sex, ethnicity, ICU category, white blood cell count, red blood cell count, hemoglobin level, platelet count, albumin level, and total bilirubin. Subgroup analysis was performed by stratifying the data based on different variables, namely age (< 1 year, ≥1 year), sex (female, male), ethnicity (Han, others), white blood cell count (< 10^9^/L, ≥10^9^/L), hemoglobin level (< 90 g/L, ≥90 g/L), and albumin level (< 36 g/L, ≥36 g/L). Notably, the interaction analysis showed that there were no statistically significant interactions between RDW and age (*P* for interaction = 0.758), sex (*P* for interaction = 0.087), ethnicity (*P* for interaction = 0.538), white blood cell count (*P* for interaction = 0.164), hemoglobin level (*P* for interaction = 0.557), and albumin level (*P* for interaction = 0.663) with respect to the presence of encephalitis. These findings indicate that the association between RDW and childhood encephalitis remained consistent across different subgroups (*P* for interaction >0.05). Our study indicated that the association between RDW and childhood encephalitis remained robust across different subgroups (*P* for interaction >0.05). No significant interactions were observed in the subgroups (All *P*-value for interaction >0.05). Detailed information regarding the association between RDW and encephalitis in children can be found in Supplementary Table S2 and Figure 3. In addition, we have plotted ROC curves to assess the predictive capability of RDW for the presence of encephalitis in pediatric patients. The corresponding data is presented in Supplementary Figure S1, and the AUC for RDW was found to be 71.1% (95% CI: 67.3%−74.8%).

Furthermore, missing values of the Glucose, C-reactive, procalcitonin, Interleukin-6 and Fibrinogen. The percentages of missing values were higher than 20%. We imputed missing data of the covariates by using multiple imputations. We conducted sensitivity analysis and added glucose, C-reactive, procalcitonin, interleukin-6, and fibrinogen to the regression model, without adding the above variables. The results were still robust.

## Discussion

This cross-sectional study using the PIC database reveals an inverse linear association between RDW and childhood encephalitis in China. To our knowledge, we demonstrate for the first time that each 1% RDW increase correlates with an 18% reduction in encephalitis prevalence among Chinese children (adjusted OR = 0.82, 95% CI:0.73–0.92). This association remained significant after comprehensive adjustment, categorical transformation of RDW and subgroup analysis, indicating its potential as an accessible biomarker for pediatric encephalitis risk stratification in PICUs.

The RDW reflects the variability in the size of erythrocytes. Hematologic inflammatory markers, such as RDW, are simple, cost-effective, and readily accessible prognostic indicators for a range of conditions. Accumulating evidence suggests that RDW is associated with various conditions, including cardiovascular disease, acute respiratory distress syndrome (ARDS), Alzheimer's disease, severe COVID-19, acute kidney injury (20–28).

While Li et al. (29) identified RDW-to-albumin ratio as prognostic in adult autoimmune encephalitis. Our study provides novel evidence of RDW's specific association with childhood encephalitis in China. We employed multivariate regression analysis, which indicated a significant association between RDW and childhood encephalitis. Furthermore, even after transforming the continuous variable RDW into a categorical variable, the association between RDW and encephalitis remained robust. This suggests that RDW holds promise as a predictive factor for the onset of encephalitis in children. The significance of our study not only stems from the inclusion of a substantial number of Chinese childhood patients but also from its relevance to current clinical management practices. We believe that these factors enhance the clinical applicability of our findings.

Recent research has indicated a notable correlation between elevated RDW levels and increased hospital mortality among individuals with sepsis (30–32). In a study by Hu et al. (33). involving 80 children aged 10–14 diagnosed with orthostatic hypertension, findings demonstrated that a 1% rise in red blood cell distribution width (RDW) was linked to a 4.008 times higher likelihood of developing orthostatic hypertension (95% CI: 1.698–9.461). Unlike prior research, which mainly focused on adults of various age groups, there has been limited investigation into children. As certain serum inflammatory markers are age-specific, the relationship between age and RDW, as well as the gradual decline in various organ functions, reduced resistance to bacterial and viral infections, and increased susceptibility to illness and mortality, differ between adults and children. Consequently, findings from adult studies may not be entirely applicable to children. In our study, we analyzed a population of 10,185 children aged 0–18 years from a large pediatric database in China from 2010 to 2018. Our research revealed an inverse relationship between RDW levels and the presence of childhood encephalitis. Our findings, as is shown in Table 2 and Figure 2, indicated a significant decrease in the presence of encephalitis among children with high RDW levels compared to those with low RDW levels. Through curve fitting, stratified analyses, and subgroup analyses, we confirmed the robustness of our results. This inverse association may be attributed to the high incidence rate and mortality of neonates, as well as the gradual improvement of body immunity and adaptation to the external environment with age, leading to a significant reduction in the likelihood of illness. Similar to our findings, a study performed by Narci et al. (34). reported that lower levels of RDW are associated with patients with acute appendicitis.

The correlation mechanism between RDW and encephalitis remains unclear. Encephalitis is a neurological dysfunction related disease caused by inflammatory processes that damage brain tissue. Millán Solano et al. (35) demonstrated that Bacterial lipopolysaccharide, cytokines, and other products of inflammation, triggering a series of local immunological events that lead to damage to neurons. RDW has been demonstrated as an integrative biomarker for a multidimensional dysfunctional physiological status which reflects variations in red blood cell size (anisocytosis) (36). Emerging evidence links RDW to blood-brain barrier (BBB) disruption—a critical pathway in neuroinflammation. Mitochondrial dysfunction (Drp1-mediated) and oxidative stress in endothelial cells increase BBB permeability during sepsis-associated encephalopathy (37, 38). As RDW correlates with these pathophysiological processes (14, 32), it may indirectly signal neurovascular compromise. Increased RDW is also associated with oxidative stress and the release of cytokines in response to inflammation. Excessive inflammatory responses result in high levels of reactive oxygen species and pro-inflammatory cytokines (39). Although genetic variants associated with RDW have been linked to telomere length, ribosomal RNA, and apoptosis, the exact role of these mechanisms has yet to be confirmed (40). Understanding the biological pathways through which RDW is connected to various health outcomes could help identify potential therapeutic targets (13). Therefore, our findings suggest RDW's potential as an integrative biomarker reflecting inflammatory burden relevant to encephalitis pathogenesis. This simple and cost-effective parameter may offer valuable insights into an individual's health status, the presence of both subclinical and clinical diseases, and the ability to predict the prognosis of patients with various acute or chronic conditions. Regardless of the underlying disorder, patients with elevated or decreased RDW values should receive closer monitoring and more intensive management to improve clinical outcomes.

However, our research has the following limitations that need attention. First, as this study was retrospective in nature, there is a possibility of uncontrolled potential confounders. To address this, we performed a multifactorial regression analysis to demonstrate the robustness of our study findings. Second, due to the limitations of the PIC database, our study did not consider potential changes in these biomarkers post-treatment, which could also impact the mortality risk in pediatric patients. the study does not take into account the etiology of encephalitis, the duration of the disease, the duration and regimen of the child's treatment before admission to the intensive care unit, cerebrospinal fluid parameters, the severity of damage to the brain parenchyma and the degree of cerebral insufficiency, etc. although a relationship between RDW and the presence of childhood encephalitis was observed, the cross-sectional design of the study precludes us from establishing a causal relationship, further prospective studies are necessary to validate these findings. Third, while our cohort reflects severe encephalitis cases requiring ICU care, generalizability to outpatient or mild presentations may be limited. Despite the PIC database being smaller than the MIMIC-III (Medical Information Mart for Intensive Care III) in terms of ICU patient numbers, it remains a significant pediatric-specific, single-center database containing vital information on children admitted to critical care units at a major children's hospital in China. It serves as a valuable supplement to pediatric intensive care unit data lacking in the MIMIC-III (16). Nevertheless, given these limitations, it is essential to design and implement multi-center-controlled trials to validate our findings.

## Conclusion

In conclusion, our study demonstrates a significant inverse correlation between RDW and the prevalence of encephalitis in Chinese pediatric patients. While providing novel clinical evidence for RDW's potential as an accessible biomarker, future prospective studies should elucidate the underlying pathophysiological mechanisms and establish causality.

## Data Availability

The analyses used the publicly available Pediatric Intensive Care (PIC) database. Access to the PIC database requires completion of the Collaborative Institutional Training Initiative (CITI) course and successful completion of the “Conflicts of Interest” and “Data or Specimens Only Research” modules to obtain permission to access the data. De-identified data underlying the results are available to qualified researchers on reasonable request to the corresponding author for the purpose of reproducing the results or replicating the procedure.

## Supplementary material

Supplementary Figure S1 Receiver operating characteristic (ROC) curves of the RDW for predicting childhood encephalitis.

Supplementary Table S1 Univariate analysis for the presence of encephalitis.

Supplementary Table S2 Stratification analysis on the association of RDW and presence of encephalitis.

Supplementary Table S3 Covariate screening between RDW and childhood encephalitis.

Supplementary Table S4 Multivariate regression analysis of association between NLR, PLR, SII, SIRI and the presence of encephalitis.
