# Supplementary figures and images for: Association between red blood cell distribution width and encephalitis based on the pediatric intensive care unit database: a cross-sectional study
Source: Front Neurol. 2025 Sep 19;16:1562921. doi: 10.3389/fneur.2025.1562921 (PMC12493089; doi:10.3389/fneur.2025.1562921)

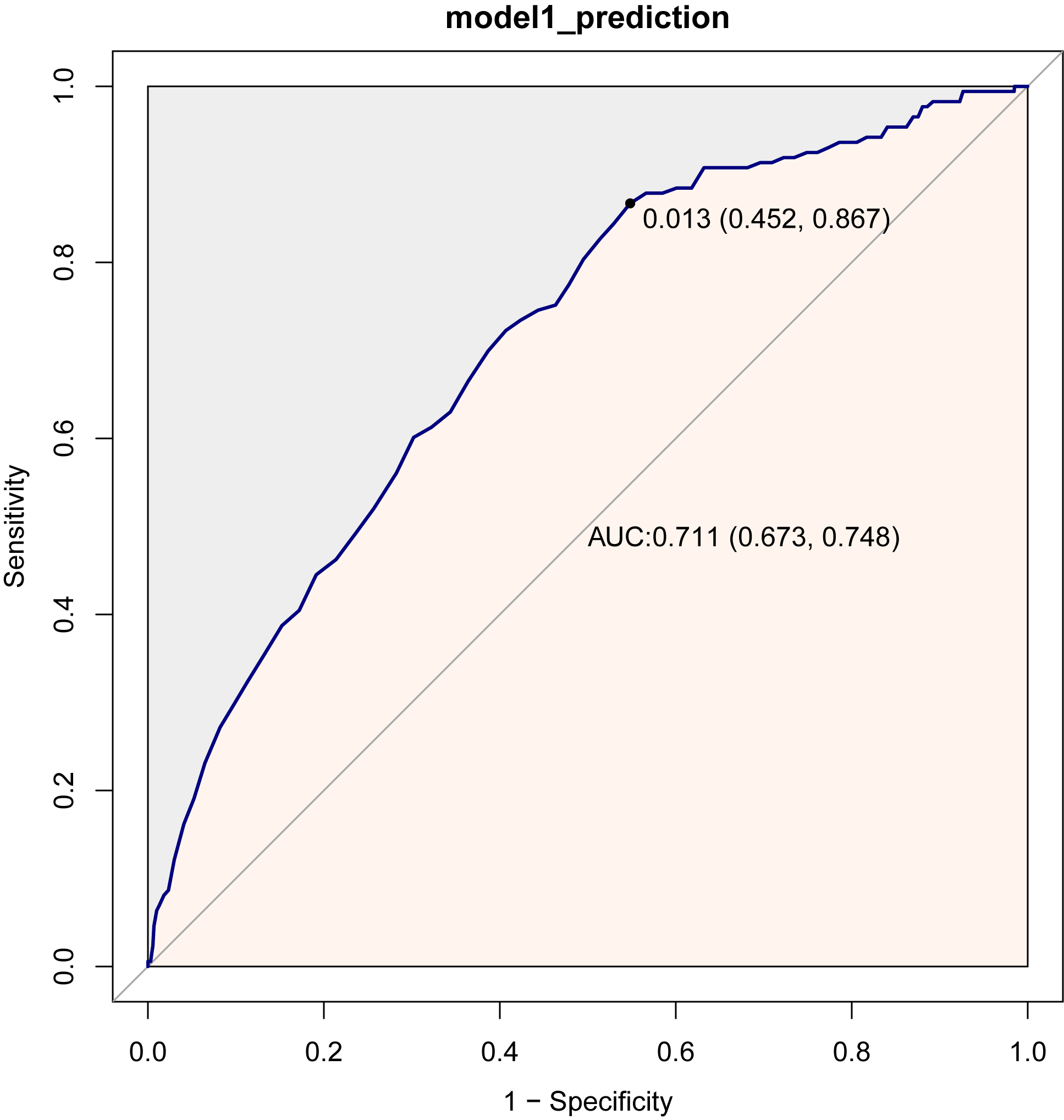

Supplement: Supplementary Figure S1 — Receiver operating characteristic (ROC) curves of the RDW for predicting childhood encephalitis. [file Image_1.tif]
