# Supplementary material for: Association between red blood cell distribution width and encephalitis based on the pediatric intensive care unit database: a cross-sectional study
Source: Front Neurol. 2025 Sep 19;16:1562921. doi: 10.3389/fneur.2025.1562921 (PMC12493089; doi:10.3389/fneur.2025.1562921)
Supplement: Supplementary Table S1 — Univariate analysis for the presence of encephalitis. [file Table_1.docx]

**Table S1. Univariate analysis for the presence of encephalitis**

| Variable | OR (95%CI) | ***P*** value |
| --- | --- | --- |
| **RDW(%)** | 0.64 (0.57~0.71) | <0.001 |
| Age(year) | 1.2 (1.17~1.24) | <0.001 |
| Sex(man), n% | 1.04 (0.76~1.4) | 0.825 |
| ethnicity(others), n% | 3.71 (1.6~8.59) | 0.002 |
| ICU category |  |  |
| GICU | 116591821.06 (0~Inf) | 0.974 |
| PICU | 163118334.4 (0~Inf) | 0.973 |
| CICU | 1 (0~Inf) | 0.999 |
| SICU | 4204285.65 (0~Inf) | 0.978 |
| WBC (10^9/L) | 0.98 (0.96~1) | 0.067 |
| RBC (12^9/L) | 1.59 (1.34~1.88) | <0.001 |
| HGB (g/L) | 1 (1~1.01) | 0.785 |
| PLT (10^9/L) | 1 (1~1) | 0.163 |
| Monocyte (10^9/L) | 0.8 (0.6~1.07) | 0.128 |
| Lymphocyte (10^9/L) | 0.59 (0.51~0.68) | <0.001 |
| Neutrophils (10^9/L) | 1.01 (0.99~1.04) | 0.308 |
| ALB (g/L) | 1.11 (1.08~1.13) | <0.001 |
| ALT(U/L) | 1 (1~1) | 0.001 |
| AST(U/L) | 1 (1~1) | 0.005 |
| Bilirubin total(µmol/L) | 0.93 (0.91~0.95) | <0.001 |
| Glucose(mmol/L) | 0.98 (0.92~1.04) | 0.456 |
| LDH(U/L) | 1 (1~1) | 0.565 |
| Potassium(mmol/L) | 0.94 (0.77~1.15) | 0.557 |
| Sodium(mmol/L) | 0.98 (0.95~1.01) | 0.229 |
| Chloride(mmol/L) | 0.92 (0.9~0.94) | <0.001 |
| Urea(mmol/L) | 1.02 (0.99~1.05) | 0.12 |
| Creatinine(µmol/L) | 1 (0.99~1) | 0.477 |
| CRP(mg/dl) | 1 (0.99~1) | 0.245 |
| PCT (ng/ml) | 1 (1~1) | 0.131 |
| IL6 | 1 (1~1) | 0.223 |
| Fibrinogen(g/L) | 1.24 (1.08~1.43) | 0.002 |
| LOS(day) | 1 (1~1.01) | 0.186 |
| Hospital LOS(day) | 0.99 (0.97~1) | 0.009 |
| hospital expire flag(mortality),n% | 2.29 (1.44~3.65) | <0.001 |
| 28dayinhosptialmortality(mortality),n% | 2.57 (1.6~4.14) | <0.001 |

Abbreviations: OR, odds ratio; CI, confidence interval. RDW: Red blood cell distribution width; CICU: cardiac intensive care unit; GICU: general intensive care unit; NICU: neonatal intensive care unit; PICU: pediatric intensive care unit; SICU: surgical intensive care unit. WBC: White blood cells; RBC: Red blood cells; HGB: Hemoglobin; PLT: Platelet; ALB: Albumin; ALT: Alanine aminotransferase; AST: Aspartate aminotransferase; LDH: Lactate dehydrogenase; CRP: C-reactive protein; PCT: procalcitonin; IL6: interleukin-6; LOS: The length of stay for the patient for the given ICU stay which may include one or more ICU units.  Hospital LOS: The length of stay for the patient for the given hospital stay. Hospital expire flag: This indicates whether the patient died within the given hospitalization.

**Table S2. Stratification analysis on the association of RDW and presence of encephalitis**

| Subgroup | Variable | n. total | n. encephalitis % | crude. OR (95%CI) | crude. ***P*** value | adj. OR (95%CI) | adj. ***P*** value | ***P***. for. interaction |
| --- | --- | --- | --- | --- | --- | --- | --- | --- |
| **Age** |  |  |  |  |  |  |  |  |
| ＜1 | RDW | 5682 | 19 (0.3) | 0.66 (0.5~0.88) | 0.004 | 0.71 (0.52~0.95) | 0.023 | 0.758 |
| ≥1 | RDW | 4503 | 154 (3.4) | 0.81 (0.73~0.91) | <0.001 | 0.86 (0.76~0.97) | 0.013 |  |
| **Sex** |  |  |  |  |  |  |  |  |
| Female | RDW | 4382 | 73 (1.7) | 0.74 (0.64~0.86) | <0.001 | 0.88 (0.76~1.02) | 0.084 | 0.087 |
| Man | RDW | 5803 | 100 (1.7) | 0.55 (0.48~0.65) | <0.001 | 0.74 (0.62~0.88) | 0.001 |  |
| **Ethnicity** |  |  |  |  |  |  |  |  |
| Han | RDW | 10083 | 167 (1.7) | 0.64 (0.57~0.71) | <0.001 | 0.82 (0.73~0.92) | 0.001 | 0.538 |
| Others | RDW | 102 | 6 (5.9) | 0.56 (0.34~0.91) | 0.019 | 0.73 (0.39~1.38) | 0.338 |  |
| **WBC** |  |  |  |  |  |  |  |  |
| ＜10^9/L | RDW | 5153 | 101 (2) | 0.61 (0.53~0.71) | <0.001 | 0.79 (0.67~0.92) | 0.002 | 0.164 |
| ≥10^9/L | RDW | 5032 | 72 (1.4) | 0.67 (0.58~0.78) | <0.001 | 0.85 (0.72~1.02) | 0.075 |  |
| **HGB** |  |  |  |  |  |  |  |  |
| ＜90g/L | RDW | 1479 | 8 (0.5) | 0.93 (0.7~1.23) | 0.614 | 0.75 (0.53~1.07) | 0.113 | 0.557 |
| ≥90g/L | RDW | 8706 | 165 (1.9) | 0.62 (0.56~0.7) | <0.001 | 0.84 (0.75~0.95) | 0.006 |  |
| **ALB** |  |  |  |  |  |  |  |  |
| ＜36 g/L | RDW | 4986 | 47 (0.9) | 0.66 (0.55~0.78) | <0.001 | 0.78 (0.65~0.93) | 0.007 | 0.663 |
| ≥36g/L | RDW | 5199 | 126 (2.4) | 0.67 (0.58~0.78) | <0.001 | 0.86 (0.75~1) | 0.05 |  |

Abbreviations: OR, odds ratio; CI, confidence interval. WBC: White blood cells; HGB: Hemoglobin; ALB: Albumin.

They were adjusted for Age, Sex, Ethnicity, ICU category, Hospital LOS, White blood cells, Red blood cells, Hemoglobin, Platelet, Albumin, and Bilirubin total.

**Table S3. Covariate screening between RDW and childhood encephalitis**

| Term1 | coeff1 | Change.percentage1 | Term2 | coeff2 | Change.percentage2 | GVIF | DF | GVIF^(1/(2*Df)) | colinearity | select | select.VIF |
| --- | --- | --- | --- | --- | --- | --- | --- | --- | --- | --- | --- |
| Crude | -0.45 | Ref. | Full | -0.09 | Ref. | 1.596 | 1 | 1.263 | 0 | Ref. | Ref. |
| **age** | -0.29 | -36.1 | age | -0.08 | -8.8 | 1.593 | 1 | 1.262 | 0 | Yes | Yes |
| gender | -0.45 | 0.8 | gender | -0.09 | 3.7 | 1.209 | 1 | 1.1 | 0 | No | No |
| ethnicity | -0.46 | 1.3 | ethnicity | -0.08 | -6.4 | 1.108 | 1 | 1.053 | 0 | No | No |
| los | -0.5 | 10.9 | los | -0.09 | -1 | 5.328 | 1 | 2.308 | 1 | Yes | Pending |
| hosptiallos | -0.45 | -0.6 | hosptiallos | -0.09 | 3.3 | 5.552 | 1 | 2.356 | 1 | No | Pending |
| hospitalexpireflag | -0.47 | 3.4 | hospitalexpireflag | -0.09 | 0.8 | 6314263 | 1 | 2512.82 | 1 | No | Pending |
| **ICUunit** | -0.31 | -31.4 | ICUunit | -0.11 | 29.6 | 2.17 | 4 | 1.102 | 0 | Yes | Yes |
| X28dayinhosptialmortality | -0.46 | 3.2 | X28dayinhosptialmortality | -0.09 | 1.9 | 6314263 | 1 | 2512.82 | 1 | No | Pending |
| wbc | -0.45 | -0.8 | wbc | -0.09 | 1.6 | 419.962 | 1 | 20.493 | 1 | No | Pending |
| **rbc** | -0.47 | 4.4 | rbc | -0.04 | -57.8 | 3.417 | 1 | 1.848 | 0 | Yes | Yes |
| **hemoglobin** | -0.48 | 7 | hemoglobin | -0.15 | 68.3 | 3.827 | 1 | 1.956 | 0 | Yes | Yes |
| platelet | -0.45 | -0.4 | platelet | -0.09 | -3.1 | 1.571 | 1 | 1.254 | 0 | No | No |
| monocyte | -0.48 | 5.7 | monocyte | -0.09 | 1.7 | 2.998 | 1 | 1.731 | 0 | No | No |
| lymphocyte | -0.39 | -12.9 | lymphocyte | -0.09 | 2.2 | 32.38 | 1 | 5.69 | 1 | Yes | Pending |
| **creactiveprotein** | -0.54 | 20.5 | creactiveprotein | -0.1 | 9.2 | 1.764 | 1 | 1.328 | 0 | Yes | Yes |
| neutrophils | -0.45 | -0.2 | neutrophils | -0.09 | 4.6 | 346.163 | 1 | 18.605 | 1 | No | Pending |
| **procalcitonin** | -0.32 | -28.9 | procalcitonin | -0.14 | 64.3 | 1.477 | 1 | 1.215 | 0 | Yes | Yes |
| **il6** | -0.33 | -26.3 | il6 | -0.07 | -18 | 1.201 | 1 | 1.096 | 0 | Yes | Yes |
| **fibrinogen** | -0.3 | -32.4 | fibrinogen | -0.08 | -13.6 | 1.701 | 1 | 1.304 | 0 | Yes | Yes |
| **albumin** | -0.37 | -16.7 | albumin | -0.08 | -6.1 | 1.761 | 1 | 1.327 | 0 | Yes | Yes |
| alanineaminotransferase | -0.45 | -0.4 | alanineaminotransferase | -0.08 | -3.7 | 13.376 | 1 | 3.657 | 1 | No | Pending |
| asparateaminotransferase | -0.45 | 0 | asparateaminotransferase | -0.08 | -11.3 | 38.897 | 1 | 6.237 | 1 | Yes | Pending |
| **bilirubintotal** | -0.26 | -42.3 | bilirubintotal | -0.09 | 2.5 | 1.333 | 1 | 1.155 | 0 | Yes | Yes |
| **glucose** | -0.48 | 6.6 | glucose | -0.23 | 155.7 | 1.46 | 1 | 1.208 | 0 | Yes | Yes |
| lactatedehydrogenase | -0.46 | 1.7 | lactatedehydrogenase | -0.09 | -1.3 | 27.93 | 1 | 5.285 | 1 | No | Pending |
| potassium | -0.46 | 2 | potassium | -0.09 | 1.9 | 1.115 | 1 | 1.056 | 0 | No | No |
| **sodium** | -0.46 | 2.2 | sodium | -0.08 | -10.8 | 2.823 | 1 | 1.68 | 0 | Yes | Yes |
| chloride | -0.44 | -3.1 | chloride | -0.09 | 1.7 | 2.673 | 1 | 1.635 | 0 | No | No |
| urea | -0.45 | 0.2 | urea | -0.09 | -0.6 | 3.374 | 1 | 1.837 | 0 | No | No |
| creatinine | -0.45 | -0.2 | creatinine | -0.09 | -2.6 | 3.201 | 1 | 1.789 | 0 | No | No |

**Table S4. Multivariate regression analysis of association between NLR, PLR, SII, SIRI and the presence of encephalitis.**

| **Variable** | **n. encephalitis %** | **Non adjusted** | |  | **Model I** | |  | **Model II** | |  | **Model III** | |
| --- | --- | --- | --- | --- | --- | --- | --- | --- | --- | --- | --- | --- |
|  |  | **OR (95%CI)** | ***P* value** |  | **OR (95%CI)** | ***P* value** |  | **OR (95%CI)** | ***P* value** |  | **OR 95%CI)** | ***P* value** |
| NLR | 173 (1.7) | 1 (1~1) | 0.579 |  | 1 (1~1) | 0.938 |  | 1 (1~1) | 0.913 |  | 1 (1~1) | 0.933 |
| PLR | 173 (1.7) | 1 (1~1) | 0.125 |  | 1 (1~1) | 0.466 |  | 1 (1~1) | 0.949 |  | 1 (1~1) | 0.801 |
| SII | 173 (1.7) | 1 (1~1) | 0.577 |  | 1 (1~1) | 0.802 |  | 1 (1~1) | 0.968 |  | 1 (1~1) | 0.83 |
| SIRI | 173 (1.7) | 1 (1~1) | 0.726 |  | 1 (1~1) | 0.85 |  | 1 (1~1) | 0.987 |  | 1 (1~1) | 0.801 |

**Abbreviations**: Q, quartiles; OR, odds ratio; CI, confidence interval; Ref: reference; NLR: neutrophil-lymphocyte ratio; PLR: platelet-lymphocyte ratio; SII: systemic immune inflammation index; SIRI: systemic inflammatory response index; RDW: Red blood cell distribution width; WBC: White blood cells; RBC: Red blood cells; HGB: Hemoglobin; PLT: Platelet.

**Model I**: adjusted by age+ gender;

**Model II**: adjusted by **Model I**+ ethnicity+ ICU category + hospital LOS;

**Model III**: adjusted by **Model II** + WBC+RBC+ HGB+PLT +Albumin +bilirubin total.
